# Prevalence of and Factors Associated with Nephropathy in Diabetic Patients Attending an Outpatient Clinic in Harare, Zimbabwe

**DOI:** 10.4269/ajtmh.15-0827

**Published:** 2017-02-08

**Authors:** Pasipanodya Ian Machingura, Vasco Chikwasha, Parmenas Nelson Okwanga, Exnevia Gomo

**Affiliations:** 1 Department of Medical Laboratory Sciences, College of Health Sciences, University of Zimbabwe, Harare, Zimbabwe.; 2 Department of Community Medicine, College of Health Sciences, University of Zimbabwe, Harare, Zimbabwe.; 3 West End Clinic, Harare, Zimbabwe.

## Abstract

There is limited information on the burden of diabetic nephropathy in developing countries. This study aimed to determine the prevalence of and factors associated with nephropathy among diabetic patients attending an outpatient clinic in Harare, Zimbabwe. In an analytical cross-sectional study, diabetic patients were consecutively enrolled and a questionnaire administered, clinical assessment conducted, and blood samples collected for human immunodeficiency virus testing and measurement of lipids, creatinine, fructosamine, and glycosylated hemoglobin levels. Urine samples were collected for determination of albumin and creatinine levels, which were used to categorize albuminuria. A total of 344 diabetic patients were enrolled. Overall, just over a third (35.8%) of patients had moderately increased albuminuria and 9.0% had severely increased albuminuria giving an overall prevalence of nephropathy of 44.8%. Prevalence of moderately increased albuminuria was slightly higher (36.5% versus 33.3%) and severely increased albuminuria slightly lower (8.8% versus 9.5%) in type 2 than type 1 diabetes patients, but the difference was not statistically significant (*P* = 0.866). Higher fructosamine and retinopathy were associated with nephropathy in both univariate and multivariate analysis {higher fructosamine (odds ratio [OR] = 1.00, confidence interval [CI] = 1.00–1.01), and retinopathy (OR = 2.80, CI = 1.64–4.97)}. We report a higher prevalence of moderately increased albuminuria and a lower prevalence of severely increased albuminuria compared with findings reported a decade ago among type 1 and type 2 diabetes mellitus patients attending the same clinic. High fructosamine and retinopathy were independent predictors of nephropathy.

## Introduction

Diabetic nephropathy is considered a major cause of end-stage renal diseases in Africa and in the developing countries.^1^ Diabetic nephropathy is defined as an increase in protein excretion in urine.^2^ The Kidney Disease Improving Global Outcomes (KDGO) 2012 guidelines classify albumin excretion as normal to mildly increased albuminuria, also referred to as normoalbuminuria; moderately increased albuminuria, also referred to as microalbuminuria; and severely increased albuminuria, also referred to as macroalbuminuria or overt proteinuria.^3^ Proteinuria and decreased glomerular filtration rate (GFR) mostly occur in parallel. However, some patients have diabetes nephropathy without increased urine albumin excretion.^2^

Racial differences in the prevalence of diabetic nephropathy and end-stage renal disease have been reported. African–American patients have a higher risk of diabetic nephropathy and kidney damage compared with Caucasians.^4^ In sub-Saharan Africa, diabetes mellitus is among the leading causes of end-stage renal disease. In a study in Egypt, diabetic nephropathy contributing to end-stage renal diseases increased from 5% in 2004 to 13% in 2007.^5^ Thus, as the burden of diabetes mellitus continues to increase, the prevalence of chronic kidney disease and end-stage renal disease will also increase.^6^

There is limited information on the burden of chronic kidney disease among diabetes mellitus patients in sub-Saharan Africa. This is partly because the screening for diabetes nephropathy is not routinely done due to limited diagnostic resources.^7^ A study conducted in 2005 in Zimbabwe reported a prevalence of nephropathy of 33% among insulin-dependent (type 1) diabetic patients. Overt proteinuria was reported in 21% of the patients and microalbuminuria in 12% of the patients.^8^

The aim of this study was to determine the prevalence of and factors associated with nephropathy among diabetic patients attending an outpatient clinic at Parirenyatwa Hospital, Harare.

## Materials and Methods

### Study design.

An analytical cross-sectional study was conducted among diabetic patients attending an outpatient clinic at Parirenyatwa Hospital, Harare, Zimbabwe, between October 23, 2013, and July 9, 2014.

### Study population.

There were 410 diabetic patients who were on regular follow-up in the outpatient clinic during the study period. All adult diabetic patients (≥ 18 years) attending the outpatient clinic at Parirenyatwa Hospital were given information about the study, and all patients who consented to participate in the study were consecutively enrolled.

### Data collection.

#### Sociodemographic data.

A questionnaire was used to collect sociodemographic data and medical history including smoking, alcohol intake, family history of diabetes mellitus, and treatment and management of diabetes.

#### Clinical data.

Weight, height, and blood pressure were measured by a research nurse. Weight was measured to the nearest kilogram using an analogue scale with the patient wearing light clothes and no shoes. Height was measured to the nearest centimeter using a stadiometer. Weight and height were used to calculate body mass index (BMI). Systolic and diastolic blood pressure were measured using a manual mercury sphygmomanometer after at least 10 minutes in a sitting position. Two readings were made and the average blood pressure was used. The absence of urinary tract infection was based on medical history and clinical examination. An ophthalmologist conducted examination for retinopathy using a slit-lamp indirect ophthalmoscopy with a diopter lens. The diagnosis of diabetic retinopathy was based on minimum of one microaneurysm in the examined field.^9^,^10^ Diabetic retinopathy was further classified as nonproliferative and proliferative diabetic retinopathy for patient management purposes.

### Laboratory investigations.

Urine and blood samples were collected for laboratory investigations. The patients were given a 100 mL sterile screw cap urine container to void a random spot midstream sample in the clinic. The patients were requested to pass a small amount of urine into the toilet, then void at least 50 mL of midstream urine sample in the urine container. Using the vacutainer method, 4 mL of blood was collected in each of ethylenediaminetetraacetic acid (EDTA) and plain tubes. Blood in the EDTA tube was used for glycosylated hemoglobin analysis and human immunodeficiency virus (HIV); blood in the plain tube was used to determine levels of fructosamine, lipids (triglycerides, total cholesterol, and high-density lipoprotein [HDL] cholesterol), and creatinine. Whole blood in EDTA tube was stored at 2–8°C, and then tested for HIV within 3 days and for glycosylated hemoglobin within 7 days of sample collection. Blood in the plain tube was centrifuged at 3,000 revolutions per minute for 5 minutes, then serum was aliquoted into cryovial tubes, which were stored at −20°C; one vial of each patient serum was thawed and analyzed for fructosamine, lipids (triglycerides, total cholesterol, and HDL cholesterol), and creatinine every 2 months. Urine sample was aliquoted into cryotubes, which were frozen at −20°C; a single vial for each patient was thawed once and analyzed for albumin and creatinine every 6 months.

Blood glycated hemoglobin, fructosamine, lipids (triglycerides, total cholesterol, and HDL cholesterol), serum creatinine, and urine creatinine were measured using an automated BS120 Analyzer (Mindray, Shenzhen, China), whereas urine albumin was measured using a BS-400 Analyzer (Mindray). The analyzers were calibrated using standards and two sets of controls supplied by the manufacturer were analyzed before samples were analyzed as per manufacturer's recommendations. Urine albumin and creatinine were used to calculate the albumin to creatinine ratio, whereas serum creatinine was used to calculate creatinine clearance. Creatinine clearance was calculated using the Chronic Kidney Disease Epidemiology Collaboration equation, based on serum creatinine concentration, age, and gender.^3^

Anonymous HIV testing was carried out using rapid (Determine, Waltham, MA; SD Bioline, Yongin-si, Gyeonggi-do, Republic of Korea; and Insti, Richmond, BC, Canada) kits following the national algorithm of HIV testing for Zimbabwe on samples in EDTA tubes for all the patients. The rapid Determine HIV test was first carried out, and only if the results were positive, a rapid SD Bioline test was used to confirm the HIV status, whereas the Insti rapid HIV test kit was the tiebreaker for any discordant result from the first two test kits.

### Definitions.

Type 1 and type 2 diabetes mellitus were categorized according to the Zimbabwe National Guidelines. Type 1 diabetes mellitus was defined as a diagnosis made usually before 30 years of age, which is treated with diet and insulin, whereas type 2 diabetes mellitus was defined as a diagnosis made after 30 years of age treated with diet and oral hypoglycemic agents with some eventually requiring insulin.^11^

Moderately increased albuminuria category (microalbuminuria) was defined as 30–300 mg albumin/g creatinine and severely increased albuminuria category (overt proteinuria) as > 300 mg albumin/g creatinine.^3^,^12^ Nephropathy was defined as moderately increased albuminuria and severely increased albuminuria.^8^ Creatinine clearance is a marker of GFR, and was defined as normal or high (≥ 90 mL/min/1.73 m^2^), mildly decreased (60 to < 90 mL/min/1.73 m^2^), mildly to moderately decreased (45 to < 60 mL/min/1.73 m^2^), moderately to severely decreased (30 to < 45 mL/min/1.73 m^2^), severely decreased (15 to < 30 mL/min/1.73 m^2^), and kidney failure (< 15 mL/min/1.73 m^2^).^3^

Hypertension was defined as a systolic blood pressure ≥ 140 mmHg and/or a diastolic blood pressure ≥ 90 mmHg, and/or taking antihypertensive medication.^13^ Controlled hypertension was defined according to the Eighth Joint National Committee criteria in diabetes mellitus patient of systolic blood pressure < 140 mmHg and diastolic blood pressure of < 90 mmHg.^14^ Uncontrolled blood pressure was defined as a systolic blood pressure of ≥ 140 mmHg and/or diastolic blood pressure ≥ 90 mmHg, elevated triglycerides ≥ 1.7 mmol/L, low HDL cholesterol < 0.9 mmol/L for male, < 1.0 mmol/L female, and obesity BMI > 30kg/m^2^.^15^ Cholesterol ≥ 5.2 mmol/L was defined as elevated.^16^

### Data analysis.

The data were captured using EPI info (Atlanta, GA) and analyzed using STATA version 13 (College Station, TX). Normally distributed data variables were summarized using mean ± standard deviation (SD), whereas nonnormally distributed data were summarized as median and interquartile range. The Student's *t* test was used to compare means for normally distributed data and the median test was used for non-normally distributed data. The Fisher's exact test or χ^2^ test was used to compare proportions. A *P* value of < 0.05 was considered statistically significant. Univariate analysis was first used to test for association of the variables with diabetic nephropathy, then all the variables that were significant (using a *P* value < 0.01) in the univariate analysis were used in the multivariate analysis using logistic regression.

## Results

A total of 348 black African diabetic patients were recruited during the study period, October 23, 2013–July 9, 2014. Four patients who did not provide a urine sample were excluded from the analysis. Of the 344 diabetic patients analyzed, 72.7% were female. Overall, mean (SD) age was 57.6 (14.8) years. There was no difference in age between males (57.0 [16.8] years) and females (57.6 [14.1] years) (*P* = 0.733). The majority of the patients (75.6%) had type 2 diabetes mellitus. None of the patients enrolled reported current history of or had signs of urinary tract infections on clinical examination. Two of the patients reported that they were smokers, whereas 14 (4.1%) reported taking alcohol. Thirty-four patients (9.9%) were HIV positive.

The mean age at diagnosis for type 2 diabetes mellitus patients was higher than that of type 1 diabetes mellitus patients: 50.7 (12.4) years versus 36.6 (13.1) years (*P* < 0.001). All type 1 diabetes mellitus patients were on insulin. Type 2 diabetes mellitus patients were on the following diabetic medications: oral hypoglycemic drugs (83.1%), oral hypoglycemic drugs and insulin (13.5%), and dietary management (3.5%). Three-quarters (75.9%) of the patients were on antihypertensive drugs. The most commonly (87.0%) used antihypertensive drugs were angiotensin-converting enzyme inhibitor or angiotensin receptor blockers. The antihypertensive drug combinations were the angiotensin-converting enzyme inhibitors enalapril (66.7% [174/261]); captopril (10.7% [28/261]); enalapril and captopril (0.8% [2/261]); and the angiotensin receptor blocker, losartan (8.8% [23/261]).

Overall mean (SD) urine albumin was 66.0 ± 112.4 mg/L, mean urine creatinine was 0.9 ± 0.6g/L, and creatinine clearance was 67.4 ± 23.1 mL/min/1.73 m^2^. Table 1 shows the prevalence of nephropathy by type of diabetes mellitus. Overall, 35.8% had moderately increased albuminuria and 9.0% had severely increased albuminuria, giving an overall nephropathy prevalence of 44.8%. Prevalence of moderately increased albuminuria was slightly higher (36.5% versus 33.3%) and severely increased albuminuria slightly lower (8.8% versus 9.5%) in type 2 than type 1 diabetes patients, but the difference was not statistically significant (*P* = 0.866).

Among HIV-positive diabetes mellitus patients, the prevalence of moderately increased albuminuria was 50% (17/34) and severely increased albuminuria 11.8% (4/34), whereas among HIV-negative diabetes mellitus patients, the prevalence of moderately increased albuminuria was 34.2% (106/310) and severely increased albuminuria was 8.7% (27/310). Among the type 1 diabetes mellitus patients, proportions of HIV-positive patients were almost similar between those with moderately increased albuminuria (14.2% [4/28]) and those with severely increased albuminuria (12.5% [1/8]). Among the type 2 diabetes mellitus patients, the proportion of the patients with HIV in moderately increased albuminuria patients (13.7% [13/95]) was similar to those in severely increased albuminuria patients (13.0% [3/23]).

Table 2 shows the classification of the patients with diabetes mellitus according to the GFR and albuminuria categories. According to the KDGO 2012 classification of risk of kidney disease based on creatinine clearance,^3^ overall, 39.8% had moderately increased to very high risk of kidney disease. The risk of kidney disease was almost twice higher (67.7%) among patients with severely increased albuminuria compared with those with normal to mildly increased albuminuria (36.6%).

Among type 1 diabetes mellitus patients, 28.6% had moderately increased to very high risk of kidney disease as classified by creatinine clearance according the KDGO 2012 guideline.^3^ The majority of the patients (39.3%) had mildly decreased GFR, whereas 32.1% normal to high, 16.7% had mild to moderately decreased, 3.6% mild to severely decreased, 7.1% had severely decreased GFR, and only 1.2% had kidney failure. Of the 8 patients who had severely increased albuminuria, 37.5% had severely decreased GFR and 12.5% had kidney failure.

According to the KDGO 2012 classification of risk of kidney disease based on creatinine clearance,^3^ overall, 43.5% had moderately increased to very high risk of kidney disease among type 2 diabetes mellitus patients. Among type 2 diabetes mellitus patients, the majority (45.8%) had mildly decreased GFR, 29.6% had mild to moderately decreased, 10.8% had moderate to severely decreased, and 2.3% had severely decreased GFR, and 0.8% had kidney failure. Of the 23 severely increased albuminuria patients, 10.4% had mild to moderately decreased, 17.9% had moderate to severely decreased, and 33.3% had severely decreased GFR.

### Predictors of diabetic nephropathy.

Table 3 shows the factors associated with overall nephropathy. In univariate analysis, nephropathy was significantly associated with lower BMI (odds ratio [OR] = 0.95, confidence interval [CI] = 0.91–0.99), longer duration of disease/years (OR = 1.03, CI = 1.01–1.05), HIV positivity (OR = 2.15, CI = 1.04–4.45), alcohol intake (OR = 0.04, CI = 0.02–0.07), higher glycosylated hemoglobin (OR = 1.20, CI = 1.10–1.31), higher fructosamine (OR = 1.00, CI = 1.00–1.01), and retinopathy (OR = 3.16, CI = 1.91–5.20). When the variables were subjected to multivariate analysis, only higher fructosamine (OR = 1.00, CI = 1.00–1.01) and retinopathy (OR = 2.80, CI = 1.64–4.97) remained significant predictors of nephropathy.

Table 4 shows the association of diabetic nephropathy with cardiovascular risk factors. Of all patients who had hypertension, 81.1% (244/301) had uncontrolled hypertension. Proportion of patients with controlled hypertension were slightly higher in patients with normal to mildly increased albuminuria (54.4%) compared with nephropathy patients (45.6%). The proportion of patients with uncontrolled hypertension was higher in normal to mildly increased albuminuria (54.1%) than in nephropathy patients (45.9%). The proportion of patients with obesity was slightly higher in normal to mildly increased albuminuria patients (25.8%) compared with nephropathy patients (21.1%). The proportion of patients with elevated triglycerides was higher in nephropathy patients (31.8%) than in those with normal to mildly increased albuminuria (22.6%). Proportion of patients with normal to mildly increased albuminuria with elevated total cholesterol (28.4%) was similar to that of nephropathy patients (30.5%). The proportion of female patients with low HDL cholesterol (30.5%) among normal to mildly increased albuminuria patients was similar to that of female patients with nephropathy (28.4%). Proportion of male patients with low HDL cholesterol (38.8%) among normal to mildly increased albuminuria patients was higher than in male patients with nephropathy (33.3%).

## Discussion

Complications of diabetes mellitus are more prevalent among African patients compared with patients in developed countries. This is attributed to late presentation and limited screening and diagnostic facilities, poor glycemic control, and inadequate treatment of complications at an early stage.^7^ In this study, we report a higher prevalence of nephropathy in type 1 diabetes mellitus patients compared with the prevalence in 2005 in the same study setting.^8^ We also observed equally high prevalence of nephropathy in type 2 diabetes mellitus patients and when all the patients were combined (type 1 and type 2 diabetes mellitus patients). The lower prevalence of diabetic nephropathy reported in 2005 in Zimbabwe could be due to exclusion of patients who had been diagnosed with renal disease, hypertension, and cardiac failure.^8^ In the present study, all diabetes patients attending the outpatient clinic at Parirenyatwa were eligible. The prevalence of nephropathy in our study is just over half of that reported in Bugando Medical Center, Mwanza, Tanzania (2011–2012).^7^ The difference could be explained by the ethnic difference between patients in Tanzania and Zimbabwe. Ethnic and racial differences in incidence and prevalence of diabetes nephropathy have been previously reported.^17^

The overall prevalence of moderately increased albuminuria and the prevalence of moderately increased albuminuria among type 1 and type 2 diabetes mellitus patients in our study were almost more than twice higher than the 12% reported in the previous study in Zimbabwe.^8^ Further, the prevalence in the present study was higher compared with that found in Muhimbili outpatient clinic, Tanzania (2003–2004), where they reported a prevalence of moderately increased albuminuria of 12.1% among type 1 and 9.8% among type 2 diabetes mellitus patients.^18^ However, the prevalence of moderately increased albuminuria in our study was lower than that found in Bugando Medical Center, Mwanza, Tanzania (2011–2012), which was 45.8%.^7^

The prevalence of severely increased albuminuria in the present study was lower than the 21% reported in 2005 in Zimbabwe.^8^ However, it was much higher than that reported in Tanzanian outpatients (2003–2004), where prevalence of severely increased albuminuria was 1.1% among type 1 and 7.2% among type 2 diabetes mellitus patients, using two 24-hour urine samples to determine albuminuria.^18^ The prevalence of severely increased albuminuria in this study was less than that found in Tanzania (2011–2012) of 34.1% in which a random urine dipstick was used to assess for albuminuria.^7^ The decrease in prevalence of moderately increased albuminuria reported in western countries has been attributed to improved metabolic control, better hypertension treatment, and use of angiotensin-converting enzyme inhibitors and angiotensin receptor blockers.^17^ The lower prevalence of severely increased albuminuria in our study could be attributed to the use of angiotensin-converting enzyme inhibitors and angiotensin receptor blockers, which was among the most commonly used antihypertensive drug combination in the present study. Angiotensin II receptor blockers combined with strict glycemic control reverses microalbuminuria and prevents the development of further renal complications.^7^

Differences in the prevalence of moderately increased and severely increased albuminuria between our study and other studies can be attributed to methodological issues, which include the differences in the method of urine collection and measurement of microalbuminuria and the patient population.^18^ In a study in Kumasi, Ghana (2007–2008), the use of strips to detect albumin concentration above 30 mg/g creatinine had a low sensitivity (37–87%) and high specificity (93–98%). The positive and negative predictive values have been reported to vary according to concentration used to define albuminuria. Hence, the monitoring of albumin in urine is recommended to be carried out using a quantitative method as was used in this study.^19^

Proteinuria which is diagnosed by albuminuria and decreased GFR indicated as creatinine clearance mostly occur in parallel.^2^ Kidney creatinine clearance < 15 mL/min/1.73 m^2^ is classified as kidney failure.^3^ In our study, prevalence of kidney failure was low, and similar to that reported in a study in Mwanza, Tanzania (0.5%).^7^

In our study, we could not differentiate whether diabetes mellitus or HIV was the cause of the nephropathy.^20^ HIV has been shown to be a strong independent predictor of nephropathy.^21^ We found an association between nephropathy and higher fructosamine and retinopathy in both univariate and multivariate analyses. Poor glycemic control is a known risk factor for the development of diabetic nephropathy. Fructosamine is a marker of prior endogenous glucose exposure over 2–4 weeks, which has been reported to be positively associated with chronic kidney disease and albuminuria.^22^ Our findings of the association between nephropathy and retinopathy are similar to those reported in Blantyre, Malawi, where albuminuria was significantly associated with retinopathy.^23^

In our study, the prevalence of the cardiovascular risk factors such as uncontrolled hypertension, obesity, elevated triglycerides, elevated cholesterol, and low HDL cholesterol were not significantly different between patients with normal to elevated albuminuria and those with nephropathy. Hypertension is the most powerful cardiovascular risk factor in the African context and has been noted as one of the greatest health challenges in Africa.^24^ The problem is worsened by lack of awareness, frequent underdiagnosis, low levels of control, and severity of its complications.^24^ The patients who do not achieve good control of blood pressure are at high risk of cardiovascular diseases, which is a major cause of mortality among diabetes mellitus patients. This is a cause of concern since approximately three-quarters of cardiovascular disease in diabetes mellitus may be attributed to hypertension.^25^ There is need for more aggressive management of hypertension in diabetes mellitus patients, taking into consideration the target of hypertension control of systolic blood pressure < 140 mmHg and diastolic blood pressure of < 90 mmHg,^14^ and that 81.1% of the diabetes mellitus patients had uncontrolled hypertension.

The prevalence of elevated triglycerides in both normal to mildly increased albuminuria and nephropathy was similar to that in the Nigeria multicenter study among diabetes mellitus patients, where they reported only 63.6% of the patients achieved triglyceride of less than 1.7 mmol/L^26^ and higher than 13% reported in southwestern Nigeria.^27^ The prevalence of elevated total cholesterol in both normal to mildly increased albuminuria and nephropathy patients was lower than 42% reported in southwestern Nigeria.^27^ Diabetes mellitus also doubles the risk of developing cardiovascular disease.^28^ The high prevalence of cardiovascular risk factors among normal to mildly increased albuminuria and nephropathy patients attending the outpatient clinic is a cause of concern, hence the need for strict monitoring of the patients to reduce the risk of cardiovascular disease.

## Conclusion

We report a higher prevalence of moderately increased albuminuria and a lower prevalence of severely increased albuminuria compared with findings reported a decade ago among type 1 and type 2 diabetes mellitus patients attending the same clinic. Prevalence of nephropathy was also higher in type 1 and type 2 diabetes mellitus patients than previously reported. Higher fructosamine and retinopathy were independent predictors of nephropathy. A significant proportion of the patients had uncontrolled hypertension, obesity, and abnormal lipids, which are risk factors for cardiovascular disease.

## Figures and Tables

**Table 1:** Prevalence of nephropathy by type of diabetes mellitus

Diabetes mellitus	*N*	Albuminuria		
		Moderately increased (%)	Severely increased (%)	Nephropathy (%)
Type 1	84	28 (33.3)	8 (9.5%)	36 (42.9)
Type 2	260	95 (36.5)	23 (8.8%)	118 (45.4)
Total	344	123 (35.8)	31 (9.0)	154 (44.8)

**Table 2:** Albumin-to-creatinine ratio and creatinine clearance by nephropathy status

Albuminuria categories	*N*	GFR categories					
		Normal or high (≥ 90 mL/min/1.73 m^2^)	Mildly decreased (60 to < 90 mL/min/1.73 m^2^)	Mild to moderately decreased (45 to < 60 mL/min/1.73 m^2^)	Moderate to severely decreased (30 to < 45 mL/min/1.73 m^2^)	Severely decreased (15 to < 30 mL/min/1.73 m^2^)	Kidney failure (< 15 mL/min/1.73 m^2^)
Normal to mildly increased	190	38 (20.0%)	81 (42.6%)	55 (29.0%)	12 (6.3%)	4 (2.1%)	0
Moderately increased	123	15 (12.2%)	63 (51.2%)	26 (21.1%)	14 (11.4%)	3 (2.4%)	2 (1.6%)
Severely increased	31	2 (6.5%)	8 (25.8%)	10 (32.3%)	5 (16.1%)	5 (16.1%)	1 (3.2%)
Nephropathy	154	17 (11.0%)	71 (46.1%)	36 (23.4%)	19 (12.3%)	8 (5.2%)	3 (2.0%)

GFR = glomerular filtration rate. Normal to mildly increased albuminuria category was defined as < 30 mg albumin/g creatinine, moderately increased albuminuria category as 30–300 mg albumin/g creatinine, and severely increased albuminuria category as > 300 mg albumin/g creatinine. Creatinine clearance was defined as normal or high (≥ 90 mL/min/1.73 m^2^), mildly decreased (60 to < 90 mL/min/1.73 m^2^), mild to moderately decreased (45 to < 60 mL/min/1.73 m^2^), moderate to severely decreased (30 to < 45 mL/min/1.73 m^2^), severely decreased (15 to < 30 mL/min/1.73 m^2^), and kidney failure (< 15 mL/min/1.73 m^2^).

**Table 3:** Logistic regression analysis of factors associated with overall nephropathy

Variable	Normal to mildly increased albuminuria	Nephropathy	Univariate analysis		Multivariate analysis	
			OR (95% CI)	*P* value	OR (95% CI)	*P* value
Sex						–
Female	141	109	1		NA	
Male	49	45	1.19 (0.74–1.91)	0.478		
Age/years, mean (SD)	56.6 (15.0)	58.9 (14.4)	1.01 (1.00–1.03)	0.144	NA	–
Body mass index, kg/m^2^, mean (SD)	27.2 (5.5)	25.7 (4.9)	0.95 (0.91–0.99)	0.011	NA	–
Duration of disease/years, mean (SD)	8.9 (9.6)	12.1 (11.3)	1.03 (1.01–1.05)	0.006	1.02 (1.00–1.05)	0.058
HIV						
Positive	13	21	2.15 (1.04–4.45)	0.039	NA	–
Negative	177	133	1			
Consume alcohol						
Yes	10	4	0.04 (0.02–0.07)	< 0.001	0.29 (0.07–1.18)	0.084
No	180	150	1	–	1	–
Taking alternative medicine						
Yes	15	11	0.90 (0.40–2.01)	0.793	NA	–
No	175	143	1			
HbA1c/%, median (IQR)	7.7 (6.3–9.7)	8.7 (7.4–11.0)	1.20 (1.10–1.31)	< 0.001	1.04 (0.91–1.19)	0.588
Fructosamine/μmol/L	321.6 (97.6)	383.1 (132.8)	1.00 (1.00–1.01)	< 0.001	1.00 (1.00–1.01)	0.009
Triglycerides/mmol/L	1.2 (0.8–1.6)	1.8 (1.1–2.1)	1.24 (0.98–1.57)	0.075	NA	–
Total cholesterol/mmol/L	4.5 (3.6–5.3)	4.5 (3.6–5.5)	1.08 (0.93–1.24)	0.328	NA	–
HDL cholesterol/mmol/L	1.1 (0.9–1.4)	1.3 (1.1–1.5)	1.60 (0.92–2.79)	0.094	NA	–
Hypertension						
Yes	163	138	1.43 (0.74–2.76)	0.288	NA	–
No	27	16	1			
On antihypertension drugs						
Yes	138	123	1.50 (0.90–2.48)	0.120	–	–
No	52	31	1			
Retinopathy						
Yes	33	60	3.16 (1.91–5.20)	< 0.001	2.80 (1.64–4.79)	< 0.001
No	151	87	1		1	

CI = confidence interval; HDL = high-density lipoprotein; HIV = human immunodeficiency virus; IQR = interquartile range; OR = odds ratio; SD = standard devation.

**Table 4:** Association of diabetic nephropathy with cardiovascular risk factors

Variable	Normal to mildly increased albuminuria < 30 mg albumin/g creatinine	Nephropathy ≥ 30 mg albumin/g creatinine	Univariate analysis		Multivariate analysis	
			OR (95% CI)	*P* value	OR (95% CI)	*P* value
Hypertension						
Controlled	31	26	0.99 (0.55–1.76)	0.969	NA	
Uncontrolled	132	112	1			
Body mass index > 30 kg/m^2^ (obesity)						
Yes	49	32	0.77 (0.46–1.27)	0.307	NA	
No	141	120	1			
Elevated (≥ 1.7 mmol/L) triglycerides/mmol/L						
Yes	43	49	1.60 (0.99–2.58)	0.057	NA	
No	147	105	1			
Elevated (≥ 5.2 mmol/L) total cholesterol/mmol/L						
Yes	54	47	1.11 (0.69–1.76)	0.671	NA	
No	136	107	1			
Female (< 1.0 mmol/L) low HDL cholesterol/mmol/L						
Yes	43	31	0.91 (0.52–1.57)	0.724	NA	
No	98	78	1			
Male (< 0.9 mmol/L) low HDL cholesterol/mmol/L						
Yes	19	15	0.79 (0.34–1.84)	0.584	NA	
No	30	30	1			

CI = confidence interval; HDL = high-density lipoprotein; OR = odds ratio.
